# Chitosan–PVA–PVP/nano-clay composite: a promising tool for controlled drug delivery

**DOI:** 10.1039/d4ra02959c

**Published:** 2024-05-15

**Authors:** Mohsin Ali, Sadullah Mir, Leonard I. Atanase, Obaid-Ur-Rahman Abid, Mohsin Kazi

**Affiliations:** a Department of Chemistry, COMSATS University Islamabad Pakistan; b Department of Chemistry, Hazara University Mansehra Pakistan; c Faculty of Medicine, “Apollonia” University of Iasi Pacurari Street, No. 11 700511 Iasi Romania leonard.atanase@univapollonia.ro; d Academy of Romanian Scientists 050045 Bucharest Romania; e Department of Pharmaceutics, College of Pharmacy, King Saud University PO BOX 2457 Riyadh 11451 Kingdom of Saudi Arabia mkazi@ksu.edu.sa

## Abstract

In this study, chitosan, polyvinyl alcohol (PVA), and polyvinyl pyrrolidone (PVP) were used to create ternary blends reinforced with organically modified montmorillonite nanoclay. Tramadol was used as a model drug to assess the efficacy of these ternary blends as drug delivery systems. The current work demonstrated the highly controlled release of tramadol *via* transdermal administration. The results of the FTIR investigation revealed the compatibility of the blending components. Among non-drug-loaded formulations, MC6 is the most stable with a 17.6% weight residue at 505 °C and MC11 is the most stable of all the drug-loaded and non-drug-loaded formulations with a weight residue of 22.0% at 505 °C. The XRD studies of the prepared formulations showed crystalline behavior. However, the SEM analysis revealed that no gaps or mixing components were uniformly dispersed in the nanocomposites. Pharmaceutical tests, such as swelling, dissolution, and permeation rates, revealed a strong influence of the PVA concentration. There was a uniform distribution of drug throughout the films with maximum encapsulation efficiency found for MC7 (96.09 ± 0.31) and minimum encapsulation efficiency for MC11 (90.56 ± 0.34)%. Compared to the sodium acetate (pH 4.5) and potassium phosphate buffers (pH 6.8) the swelling and erosion were higher in hydrochloric acid buffer (pH 1.2). An increase in PVA concentration (or decrease in PVP concentration) increases the swelling, dissolution, and permeation rates. In addition, erosion increased with increasing PVP concentration. Furthermore, the nanoclay-reinforced composite showed high permeation. Based on the obtained results, it can be concluded that the produced nanocomposite could be used as an efficient transdermal drug delivery system.

## Abstract 2

Chitosan, polyvinyl alcohol (PVA), and polyvinyl pyrrolidone (PVP) were used to formulate a controlled transdermal drug delivery system for tramadol.

## Graphical abstract

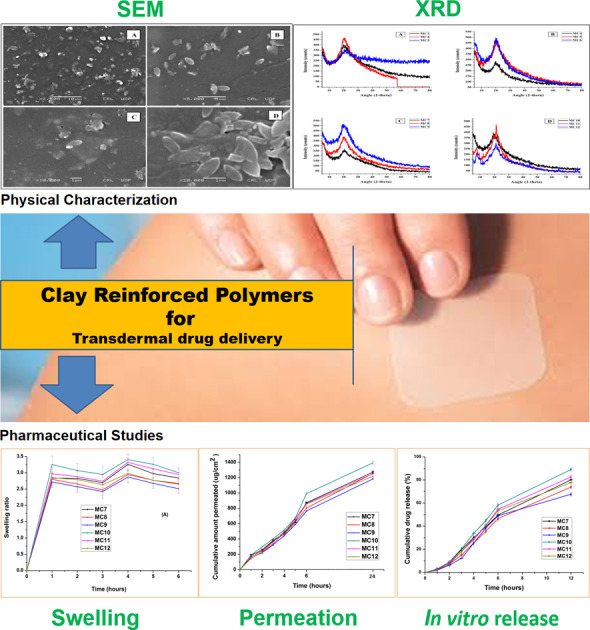

## 1 Introduction

Drug delivery systems based on nanotechnology have high health and economic output because they enable novel pharmaceutical treatments that accurately target the site of the disease, optimize the time and space of drug distribution, and help to reduce medication costs.^1^ The present work provides an improved and controlled release system for the transdermal delivery of tramadol HCl in the form of ternary blends of chitosan, PVA, and PVP.

By preventing over- and under-dosing and avoiding the liver, a transdermal drug delivery system (TDDS) is preferred over traditional drug delivery methods.^2^ Transdermal patches are used in transdermal drug delivery methods to release pharmaceuticals over a longer period of time and prevent frequent dosing. These patches are made from materials that are safe for human skin. TDDSs are excellent substitutes for conventional drug delivery systems because they have few side effects.^3^

Tramadol (HCl) is an opioid and non-opioid analgesic used to treat depression and anxiety. The fast metabolism and short retention time of tramadol are major issues. Therefore, it is necessary to design a controlled drug delivery system for tramadol HCl to meet the issues of frequent dosing.^4^ To develop such a system, biodegradable polymers are used. In TDDSs, biodegradable polymers are used because of their multiple advantages, *i.e.*, they are safe to use, and they are also eco-friendly since they degrade and do not cause any harm to the environment.

The biodegradable polymers commonly employed are polylactic acid, polyethylene glycol (PEG), alginate, chitosan, soy protein, polycaprolactone, poly-3-hydroxybutyrate, polyglycolic acid, and polyglycolide copolymers.^5^ Chitosan (CS) is the second most abundant polysaccharide in nature and is nontoxic, biocompatible, and biodegradable.^6^ It can be created synthetically from chitin and is utilized to create controlled delivery systems.^7,8^ Polyvinyl alcohol, a well-known biopolymer, is widely used in advanced biomedical applications due to its biocompatibility, reduced protein adsorption, chemical resistance, and high water solubility. It is used in artificial organs, contact lenses, dressings, and wound healing.^9^ PVA is also noncytotoxic.^10^ Polyvinyl pyrrolidone is extensively used in various fields because it is hydrophilic/polar, biocompatible, and complex.^11–13^ It is frequently used in the fields of medicine and in applications involving biological systems.^14^ The fact that CS, PVA, and PVP are biodegradable and biocompatible, as well as the fact that their ternary blends increase the cross-linking between their functional groups and prolong the drug release duration, make them attractive choices for a controlled drug release mechanism.^15–17^

Organic biodegradable polymers and organically modified clay nanoparticles are combined to create polymer–clay nanocomposites.^18^ Nanoclay serves to enhance the compatibility of immiscible polymers, thereby improving the blend properties.^19^ A substantial class of biological nanocomposites is created when clay and polymers interact atomically.^20^ A hydrated aluminosilicate clay mineral called montmorillonite (MMT) has a platelet-like structure.^21^ Reactive species are found on the surface of clay, and they interact with the medication through an ion exchange process. MMT was shown to aid in the sustained release of drugs.^22^

Any transdermal medication travels through the skin in two phases, disseminating into deeper tissues after permeating through the stratum corneum. Upon diffusion, it arrives at the desired location and performs its function. The extent and rate of drug transport are affected by ionic strength, size, hydrogen bonding, physicochemical properties, and log *P* (the partition coefficient of a molecule between aqueous and lipophilic phases).^23^ Transdermal patches may be used to address problems with traditional methods, such as under- and overdosing, as well as problems specific to tramadol, such as rapid excretion and short retention time. In the present work, a controlled-release formulation system for tramadol with minimal side effects that might be very significant to the pharmaceutical sector was reported. In this work, we investigated the effect of thin films made of chitosan–PVA–PVP nanocomposites on targeted tramadol delivery. Thermogravimetric analysis (TGA), X-ray diffraction (XRD), scanning electron microscopy (SEM), and Fourier transform infrared (FTIR) spectroscopy were used to evaluate the structure and morphology of the nanocomposite films, and pharmaceutical tests, including those on swelling and erosion, water content and homogeneity of drug content, penetration through rat skin, and dissolution, were also carried out.

## 2 Materials and methods

### 2.1. Materials

Chemicals that were pure and distilled were purchased from multiple vendors. Chitosan, polyvinyl alcohol, polyvinyl pyrrolidone, KCl, acetic acid, NaOH, glycerol, and nanoclay were procured from Sigma-Aldrich. KH_2_PO_4_ and sodium acetate were obtained from Daejung (South Korea) and Merck (Germany), respectively, while tramadol was obtained from Global Pharmaceuticals (Islamabad, Pakistan). The Comsats University Abbottabad Campus supplied distilled water.

### 2.2. Preparation of chitosan–PVA–PVP nanocomposites

The solvent casting approach was used to synthesize the CS–PVA–PVP nanocomposites, with minor modifications to the previously described process.^24^ With steady stirring, chitosan, PVA, PVP, and glycerol (as plasticizer) were added and dissolved in water. After 15 minutes of regular stirring and nanoclay addition to the polymeric solution, tramadol was added. A homogeneous solution was obtained after 30 minutes of agitating the mixture at 60 °C. After fully dissolving, the solution was transferred to Petri dishes and dried for 24 hours at 50 °C in an oven. Drying produced thin films. Twelve distinct formulations, as shown in the formulations table below (Table 1), were created by adjusting the concentrations of the PVA, PVP, nanoclay, and tramadol.^25^ There are four different sets of formulations, *i.e.* the samples MC-1 to MC-3 contain polymers only, the samples MC-4 to MC-6 contain nanoclay and polymers to study the effect of nanoclay, the samples MC-7 to MC-9 contain polymers, nanoclay and drug to study the effect of nanoclay on drug release and samples MC-10 to MC-12 contain polymers and drug to study the effect of polymers on drug release.

**Table 1:** The composition of the different polymers in the formulation

Sample	Chitosan (g)	PVA (g)	PVP (g)	MMT (g)	Tramadol (g)	Glycerol (g)
MC-1	2.5	1.875	0.625	0	0	1.25
MC-2	2.5	1.25	1.25	0	0	1.25
MC-3	2.5	0.625	1.875	0	0	1.25
MC-4	2.5	1.25	1.25	0.075	0	1.25
MC-5	2.5	1.25	1.25	0.15	0	1.25
MC-6	2.5	1.25	1.25	0.25	0	1.25
MC-7	2.5	1.875	0.625	0.075	0.375	1.25
MC-8	2.5	1.25	1.25	0.075	0.375	1.25
MC-9	2.5	0.625	1.875	0.075	0.375	1.25
MC-10	2.5	1.875	0.625	0	0.375	1.25
MC-11	2.5	1.25	1.25	0	0.375	1.25
MC-12	2.5	0.625	1.875	0	0.375	1.25

### 2.3. Characterization of formulations

The following characterization tests were performed on the prepared formulations.

#### 2.3.1. FTIR analysis

For the structure determination of prepared formulations, Fourier Transform Infrared spectroscopy (Thermo Scientific Nicolet 6700 USA) was used. After grinding, the synthesized films were mixed with KBr, and the spectra were scanned between 4000 and 500 cm^−1^.^25,26^

#### 2.3.2. Thermal analysis

Shimadzu DTG-60H NG12 5AW Thermal Analyzer (Nottinghamshire, the United Kingdom) was used to analyze the thermal stability of the prepared films. The flow of N_2_ gas through the samples in the analytical pan was approximately 20 ml min^−1^. After placing approximately 4 mg of sample in an aluminum pan, the samples were permitted to undergo thermal breakdown at 0–600 °C such that the weight loss as the temperature rose could be continually monitored.^25,27^

#### 2.3.3. XRD

The type of the produced films—crystalline or amorphous—was determined *via* X-ray diffraction analysis. The samples were analyzed using an X-ray diffractometer (Philips XPERT PRO 3040/60) across a 2*θ* range of 5–90°.^25,28^

#### 2.3.4. Scanning electron microscopy

A JSM 6400F scanning electron microscope was used to study the morphology of the synthesized formulations. The chosen voltage range was 5–15 kV. The sample was gold-coated after being placed on an aluminum holder.^25,29^

#### 2.3.5. Energy-dispersive X-ray

To perform EDX analysis, the (JSM 6400F SEM; Jeol) scanning electron microscope was used. The sample was coated with gold after being placed on an aluminum holder. The EDX procedure was completed at a voltage of 20.194 kV. The composition of the matrix, as well as the purity of the mixture components, was determined using EDX.^25,28^

### 2.4. Preliminary tramadol solubility experiment

Solubility tests were carried out with several solvents. First, 50 ml of various solvents was used to dissolve the tramadol. For 24 hours, the solutions were stirred at 37 ± 0.5 °C, and then the excess medication was removed by centrifuging the mixtures. Following suitable filtering, the residue layer was diluted with the appropriate solvents, and the tramadol content was determined at 218 nm.^25,30^

### 2.5. Calibration curve plot

To determine the amount of tramadol used in subsequent studies, a conventional calibration curve was generated. Tramadol (100 mg) was diluted in a buffer solution of KH_2_PO_4_ at pH 6.8 to create a stock solution (100 ml). By varying the concentration of the tramadol solution to between 2 and 20 g ml^−1^, 9 distinct solutions were prepared. Using KH_2_PO_4_ buffer with a pH of 6.8 as a reference, these solutions were examined at 218 nm.^25,31^

### 2.6. Drug content uniformity test

Thirty milligrams of the sample was dissolved in a potassium phosphate buffer solution with a pH of 6.8 in a 100 millilitre volumetric flask. The sample solution was stirred continuously for 24 hours. After 24 hours, sample aliquots were obtained and diluted with potassium-phosphate buffer (pH 6.8) to assess UV absorption at 218 nm.^25,31^

### 2.7. Swelling studies

For swelling investigations, buffer solutions containing potassium phosphate (pH 6.8), sodium acetate (pH 4.5), and hydrochloric acid (pH 1.2) were used. Simultaneously, 30 ml of buffer was used to dissolve 30 mg of each sample. At 1, 2, 3, 4, 5 and 6 hour intervals, the patch was removed, the excess buffer was removed, and the sample weight was recorded. The following formulae were used to calculate the swelling ratio (SR) and the percentage of water content (%):^25,32^

1 SR = *W*_s_/*W*_d_

2 Percent water content (%) = [*W*_s_ − *W*_d_/*W*_s_] × 100,

where *W*_d_ and *W*_s_ are the weights of the dry and swollen films, respectively.

### 2.8. Erosion studies

Swollen films were used in erosion studies, *i.e.*, they were individually weighed (PA214; Ohaus Corporation) following 20 minutes of drying at 50 °C in an oven. Until a constant weight was achieved, readings were made in triplicate every one, two, three, four, five and six hours. At different time intervals, film erosion (%) was calculated using the following equation:^25,33^

3 Film erosion (%) = [(*W*_0_ − *W*_2_)/*W*_0_] × 100,

*W*_0_ and *W*_2_ represent the initial wet and ultimate dry film weights, respectively.

### 2.9. Rat skin preparation

Eighteen Sprague-Dawley rats, each with a mass index of 200–250 g, were provided by the Pharmacy Department at COMSATS University in Abbottabad, Pakistan. In this experiment, the rats were maintained in an environment with alternating cycles of light and darkness.^25,34^ The anesthetic used was chloroform. The abdominal skin was first shaved using manual blades, and then the epidermis was scraped off. After cleaning the dermal fat, the skin was put in a 0.9% brine solution to eliminate debris and enzymes. The skin was cleansed with disinfected water, coated with aluminum foil, and kept at 20 °C until use. In preparation for the experiment, the Franz diffusion compartments were used to position the frozen excised rat skin, with the epidermal part facing the donor cell and the other part facing the recipient chamber.^23,25^ All study protocols were approved by the Departmental Research Ethics Committee of COMSATS University Islamabad with approval number 893/CUI/PHM-2022.

#### 2.9.1. Permeation analysis

A Franz diffusion apparatus was used.^25,35^ KH_2_PO_4_ buffer, pH 6.8, was poured into the receptor area of the diffusion cell to the point where it contacted the rat's skin. Clamps held the compartments together while the skin was attached between them. In the experiment, a weighed amount (40 mg) containing 2.25 mg of the drug was used. To prevent drying, the sample was applied to rat skin and covered with an aluminum sheet. Readings were recorded at intervals of 1, 2, 3, 4, 5, 6, and 24 hours using tiny serial dilutions of the sample withdrawn from the receptor part, and the same quantity of buffer was added. The temperature was maintained at 37 ± 0.5 °C. Tramadol concentration measurements of the samples were performed at 218 nm.^25^

### 2.10. Dissolution studies

A previously published approach was modified somewhat to perform a triplicate dissolution experiment.^36^ For the dissolution experiment, 100 mg of the nanocomposite film was dissolved in 250 ml of potassium phosphate buffer (pH 6.8). The buffer solution containing the sample was stirred with a magnetic stirrer at 100 rpm in a beaker, and the temperature was maintained at 37 ± 0.5 °C for 12 hours. As 5 ml samples were taken for analysis, the solubility media were replaced at intervals of 1, 2, 3, 4, 5, 6, and 12 hours for an equivalent volume of fresh buffer. An O.R.I. 3000, UV-visible spectrophotometer, was utilized for analysis to measure the percentage of drug release at 218 nm.^25^

### 2.11. Statistical test

For the statistical analysis, GraphPad Prism 9.4.0 was used (https://www.graphpad.com/).

## 3 Results and discussion

### 3.1. Fourier transform infrared (FTIR) spectroscopy

One of the most popular techniques for obtaining useful data on the relationships between functional groups is FTIR spectroscopy. Using FTIR analysis, the structural changes of the different functional groups in the produced formulations have been studied.^37,38^

**Fig. 1:**
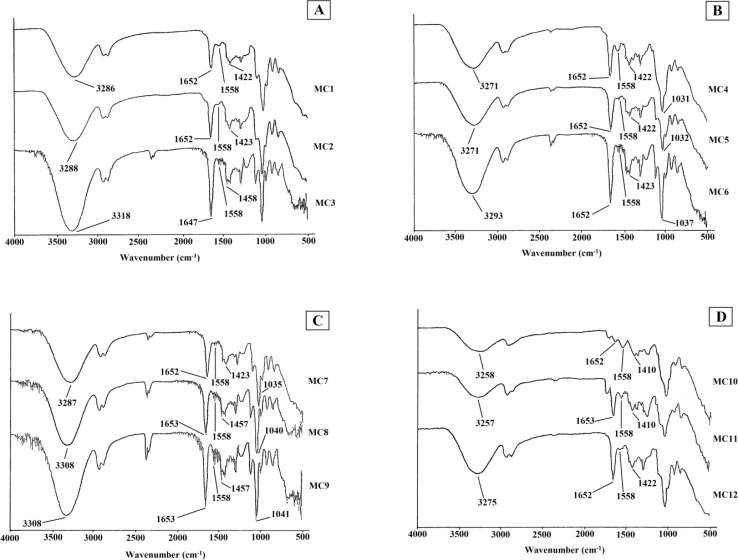
FTIR spectra of (A) MC1–MC3, (B) MC4–MC6, (C) MC7–MC9, and (D) MC10–MC12 chitosan–PVA–PVP nanocomposite films.

FTIR was utilized to detect various functional groups in the prepared formulations (Fig. 1). Several vibrational modes for OH, C=O, N–H, C–O–C, Si–O–Si, C–N, and C=N in nanoclay, PVA, and tramadol (drug) were investigated.

According to the literature, the OH group appears at 3337 cm^−1^ in pure chitosan and PVA^39,40^ and at 3550–3200 cm^−1^,^41,42^ while it appears at 3288, 3286, and 3318 cm^−1^ in samples MC1, MC2, and MC3, respectively. The decrease in the frequency shift may be attributed to hydrogen bonding (both intermolecular and intramolecular H-bonding) and demonstrates the compatibility of the mixed polymers. OH groups can be found in the drug-containing samples MC10, MC11, and MC12 at 3258, 3257, and 3275 cm^−1^, respectively, and exhibit the same pattern of frequency decrease as indicated in MC1–MC3. The C=O groups of chitosan and PVP appear at 1648 (ref. 43) and 1660 cm^−1^,^44^ respectively, whereas those of MC1, MC2, and MC3 appear at 1652, 1652, and 1647 cm^−1^, respectively. C=O was observed at 1652, 1653, and 1652 cm^−1^ in the drug-containing formulations MC10, MC11, and MC12, respectively. Lawrie *et al.* observed the N–H band of pure chitosan at 1594 cm^−1^, whereas it appeared at 1558 cm^−1^ in all nondrug-loaded (MC1, MC2, and MC3) and drug-loaded (MC10, MC11, and MC12) samples. The OH group emerges at 3271, 3271, and 3293 cm^−1^ in the nondrug-loaded nanoclay samples (polymer nanocomposites) MC4, MC5, and MC6, demonstrating a similar pattern as that observed for the basic composites (MC1–MC3) and drug-loaded nanocomposites (MC7–MC9). The C=O groups for MC4, MC5, and MC6 emerged at 1652 cm^−1^, but those for pure chitosan and PVP appeared at 1648 and 1660 cm^−1^, respectively. The N–H band emerges at 1558 cm^−1^ for MC4, MC5, and MC6 with a much lower frequency shift than that of pure chitosan N–H (1580 cm^−1^), indicating high compatibility. According to Andrés *et al.*, pure nanoclay has a Si–O–Si stretching frequency between 1068 and 1000 cm^−1^.^45^ The Si–O–Si group appears at 1031, 1032, and 1037 cm^−1^ in the nondrug-loaded nanocomposites MC4, MC5, and MC6, respectively, while it appears at 1035, 1040, and 1041 cm-1 in the drug-loaded formulations MC7, MC8, and MC9, respectively. The C–N peaks of pure tramadol and PVP appear at 1439^46^ and 1495 cm^−1^,^47,48^ respectively. It appears at 1423, 1457, and 1457 cm^−1^ for the drug-loaded nanocomposites MC7, MC8, and MC9, respectively. The peak at 1410, 1410, and 1422 cm^−1^ was attributed to the MC10, MC11, and MC12 drug-loaded composites, respectively.

The detected wave-numbers showed good interaction in each formulation, indicating that these formulations are stable enough to be used as promising future drug delivery tools.

### 3.2. Thermogravimetric analysis

**Fig. 2:**
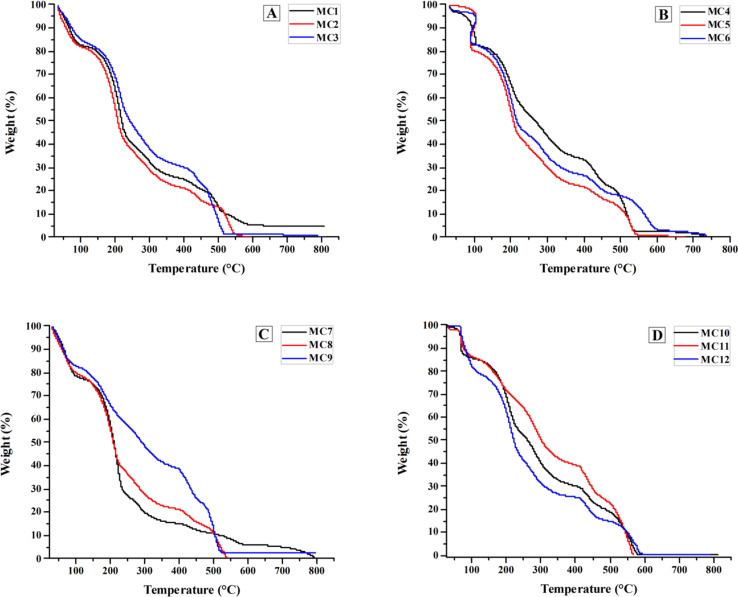
TGA thermograms of (A) MC1–MC3, (B) MC4–MC6, (C) MC7–MC9, and (D) MC10–MC12 chitosan–PVA–PVP nanocomposite films.

TGA was used to determine the thermal stability of the samples (Fig. 2). The prepared formulations were categorized into four types based on their composition. MC1–MC3 are composites consisting solely of polymers (PVA, PVP, and chitosan), whereas MC4–MC6 are nanocomposites that also contain nanoclay. MC7–MC9 contained polymers, nanoclay, and drugs, whereas MC10–MC12 contained only drugs and polymers (no nanoclay).

Among the nondrug-loaded composites MC1–MC3, samples MC1 and MC2 had similar stabilities, with weight residues of 11.7% and 12.4%, respectively, at 505 °C, while MC3 had a weight residue of 4.7%. Among the MC4–MC6 (nondrug-loaded nanocomposites), MC6 is the most stable, with a weight residue of 17.6%. A comparison of MC3 and MC6 revealed that the nanoclay improved the thermal stability of MC6. At 505 °C, MC4 and MC5 had 15.5% and 11.7% weight residues, respectively. The residue weights of the MC10, MC11, and MC12 drug-loaded composites were 18.0%, 22.0%, and 14.0%, respectively. Compared with the nondrug-loaded composite MC1–MC3, tramadol (drug) improved the thermal stability of these samples. At 505 °C, the weight fractions of the MC7, MC8, and MC9 samples containing both nanoclay and drug were 10.5%, 9.3%, and 10.4%, respectively. They are less stable than MC4–MC6 and MC10–MC12, indicating that the drug and nanoclay have no positive impact on the thermal stability when present together. Among all formulations, MC11 is the most stable, with a residual weight of 22.0% at 505 °C.

Despite their different concentrations, the TGA behavior of the prepared formulations showed an almost semilinear decrease in total weight. The TGA curves of the samples show two major phases of weight loss over two temperature ranges: room temperature to approximately 550 °C and 550 to 800 °C. The first phase of weight loss occurs by the loss of physisorbed water up to 200 °C, followed by the major degradation of PVA (water, aldehydes, ketone, alkanes, and alkenes) up to 380 °C. The third portion of weight loss began at 380 °C and was attributed to the breakdown of PVP, which has a higher temperature stability than PVA. The critical temperature for all investigated formulations was approximately 550 °C, at which all samples began exhibiting complete decomposition during the second stage of weight loss.^38,49^

### 3.3. X-ray diffraction (XRD)

XRD was used to determine whether the prepared formulations were crystalline or amorphous.

**Fig. 3:**
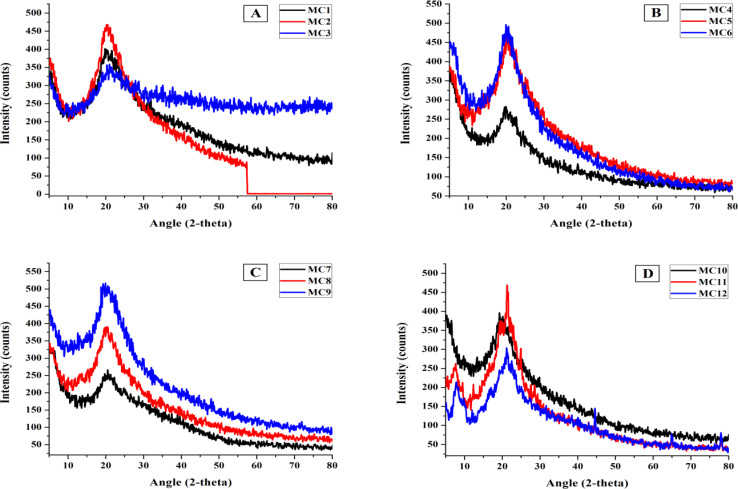
XRD patterns of (A) MC1–MC3, (B) MC4–MC6, (C) MC7–MC9, and (D) MC10–MC12 chitosan–PVA–PVP nanocomposite films.

The samples contained polymers (CS, PVA, and PVP), nanoclay, and tramadol. Feng *et al.* reported chitosan at 2*θ* = 10.6°, 11.4°, 20.1°, and 20.4°,^50^ Ricciardi *et al.* reported PVA at 2*θ* = 19.4° and 20°,^51^ and Kumar *et al.* and Li *et al.* reported PVP at 2*θ* = 13° and 21°.^52,53^ Given that the peaks of all the polymers (chitosan, PVA, and PVP) appear at approximately 20°, it is difficult to distinguish between them. The spectra of the prepared formulations exhibit intense sharp peaks at 20° and 21°, confirming that these polymers are crystalline (Fig. 3).

Polymers and nanoclay are mixed together in samples MC4–MC6. Nanoclay is observed at 2*θ* = 3.9°, 6.3°, 11.64°, 11.8°, and 12°, with sharp peaks at approximately 5°, 6°, and 9°, indicating that the nanoclay is present in crystalline form. In formulations MC7–MC9, both tramadol and nanoclay are distributed in polymers. The sharp peaks at approximately 2*θ* = 18°, 24°, and 26° confirmed the presence of crystalline tramadol, as Sohail *et al.* reported that pure tramadol appeared at 2*θ* = 10°, 12°, 16°, 18°, 24°, and 26°.^54^ All of the components can be found as crystalline substances. In composites containing only polymers (MC1–MC3), the peak value increased from 20° to 21°, indicating that the crystallinity of these polymers improved. Similar behavior is observed in formulations containing nanoclay and tramadol—that is, both tramadol and nanoclay enhance the crystalline behavior of polymers and show excellent affinity.

The crystalline morphology of the synthesized formulations contained only the polymers CS, PVA, and PVP, as determined by XRD. The addition of nanoclay and tramadol to the original blend structure resulted in a firmed crystal structure with a main peak at approximately 2*θ* = 20 and another peak at approximately 2*θ* = 11. Because of the many new bonds formed between the original blend structure and the additives, the presence of nanoclay and tramadol reduces the average crystallites, which is manifested as a minor change in the value of the inter-planar and inter-chain spacing due to a small difference in the angle of the blend's peak.^55^

### 3.4. Scanning electron microscopy (SEM)

**Fig. 4:**
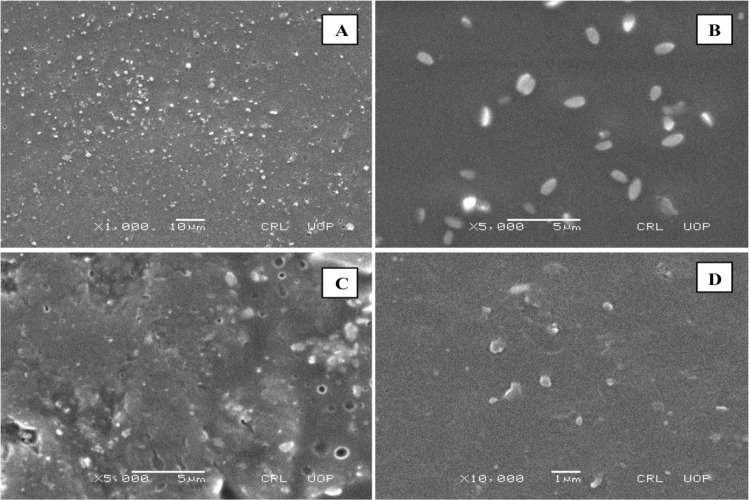
SEM images of the MC4 formulation at (A) ×1000, (B) ×5000, (C) ×5000, and (D) ×10 000 magnification.

**Fig. 5:**
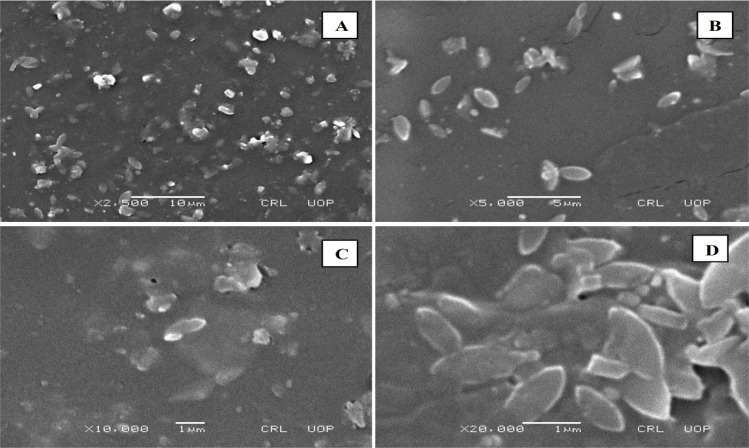
SEM images of the MC8 formulation at (A) ×2500 (B) ×5000 (C) ×10 000 (D) ×20 000 magnification.

**Fig. 6:**
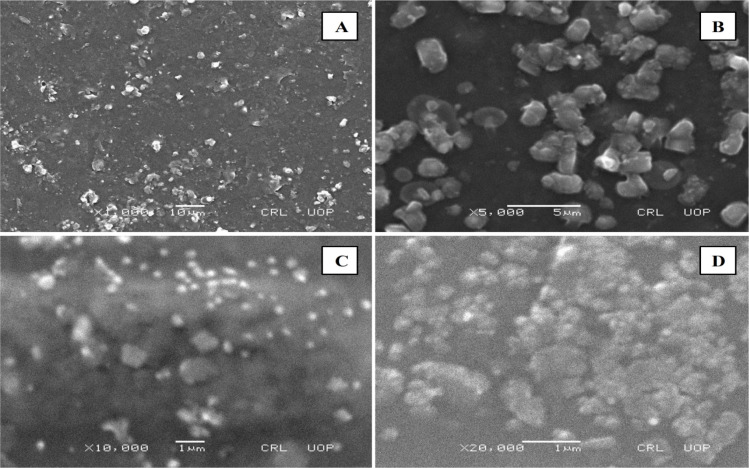
SEM images of the MC11 formulation at (A) ×2500, (B) ×5000, (C) ×10 000, and (D) ×30 000 magnification.

Scanning electron microscopy (SEM) is useful for studying particle size and morphology (Fig. 4–6). SEM was used to detect the presence and mode of nanoclay dispersion. The nanoclay was distributed evenly in the film matrix, and no gaps or cracks were found in the formulations, indicating that the nanoclay and polymer matrix were compatible. Small spots corresponding to clay nanoparticles were observed at high resolution, indicating compatibility between the nanoclay and the polymer matrix. The nanoclay had particle sizes ranging from 300 to 500 nm. This good interaction confirms the microscopic structure of the material, as shown by FTIR and XRD analysis, and is reflected in the macroscopic properties of the material.

### 3.5. Energy dispersive X-ray (EDX) spectroscopy

**Fig. 7:**
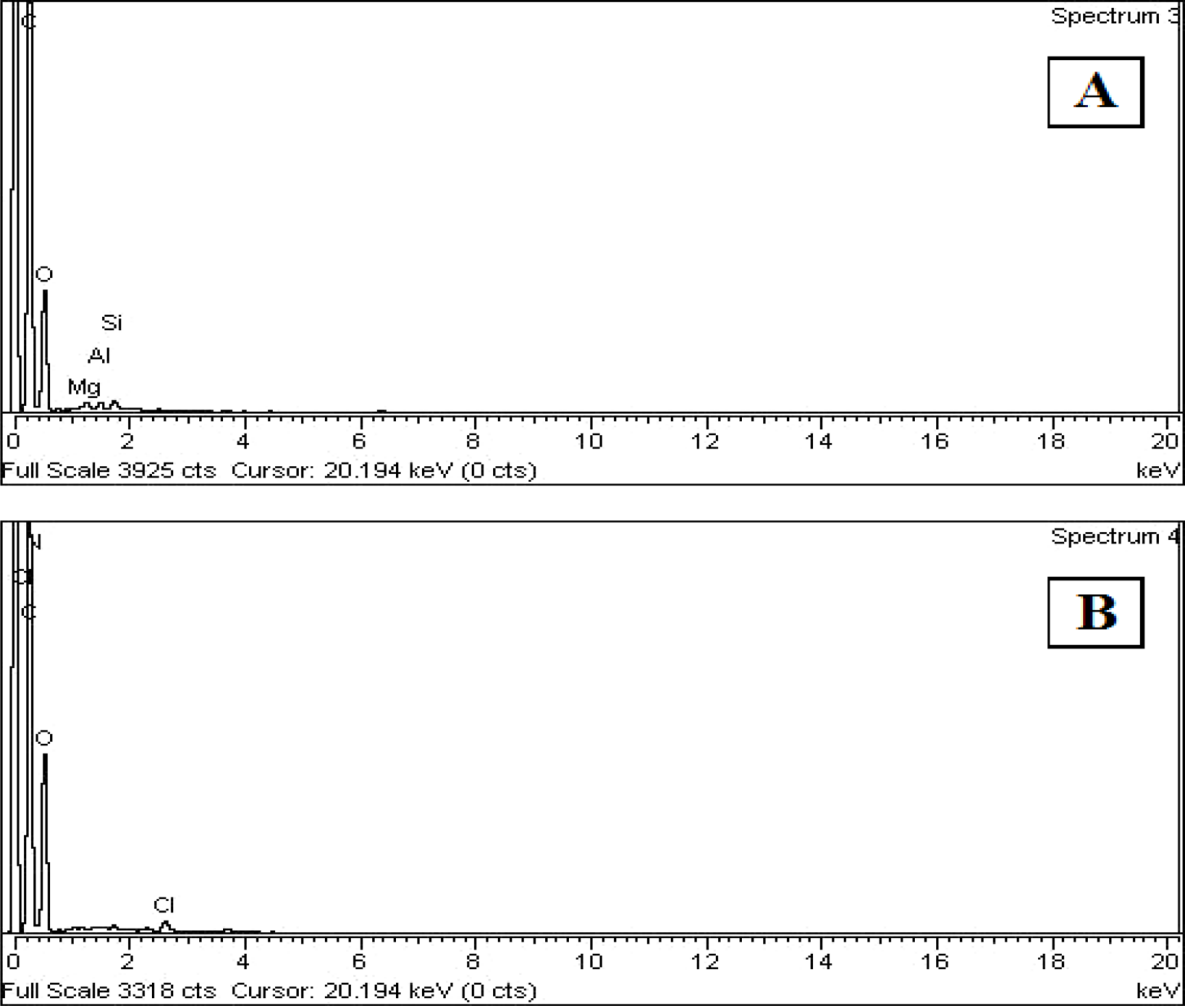
EDX profiles of formulations (A) MC8 and (B) MC11.

The use of EDX aids in determining the precise percentage of each element present in the sample (Fig. 7). EDX analysis was used to investigate the elemental composition of formulations MC8 and MC11. The sharp peaks of N, O, and C indicate that more chitosan, PVA, and PVP were present in the formulations. The presence of tramadol hydrochloride was confirmed by the presence of Cl, whereas the presence of Ca, Mg, and Si confirmed the presence of nanoclay. The last three elements were not present in sample MC11 because nanoclay was not added to the blend.

### 3.6. Method validation for tramadol·HCl concentration measurement

**Fig. 8:**
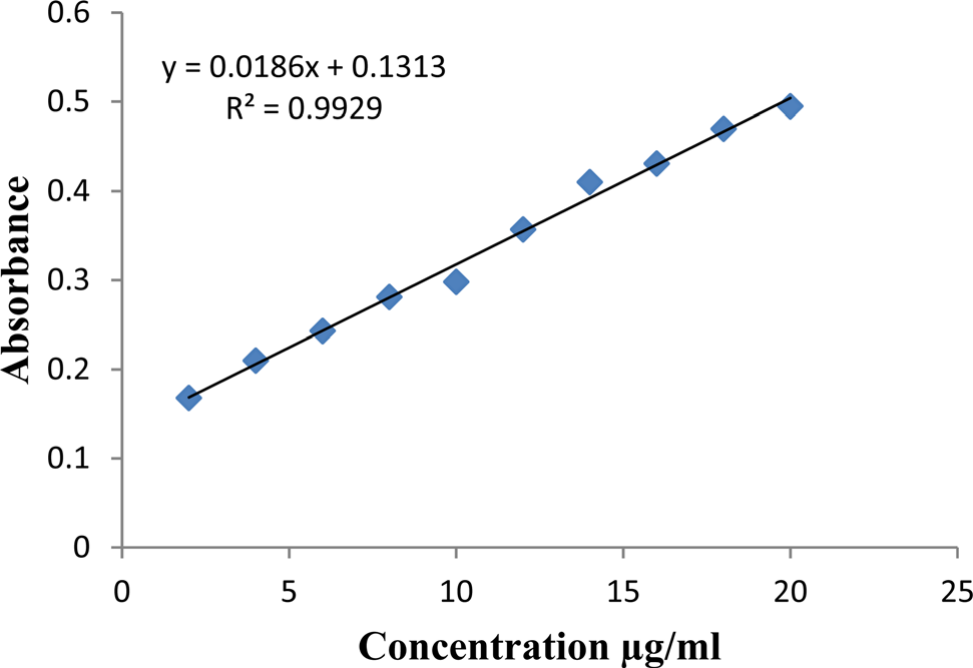
Calibration curve of tramadol.

UV-visible spectrophotometric analysis was used to validate the method for measuring the tramadol concentration (Fig. 8). First, a blank sample was run using potassium phosphate buffer solvent only. After that, the absorbance values of the different concentrations of tramadol solutions were measured. The solutions were prepared in KH_2_PO_4_ pH 6.8 buffer with a concentration range of 2–20 μg ml^−1^. The standard calibration curve for tramadol was drawn using sample absorbance at concentrations of 2, 4, 6, 8, 10, 12, 14, 16, 18, and 20 μg ml^−1^. The *Y*-equation was measured to be 0.0186*x* + 0.1313 with a determination coefficient (*R*^2^ value) of 0.9929.

### 3.7. Swelling studies

**Fig. 9:**
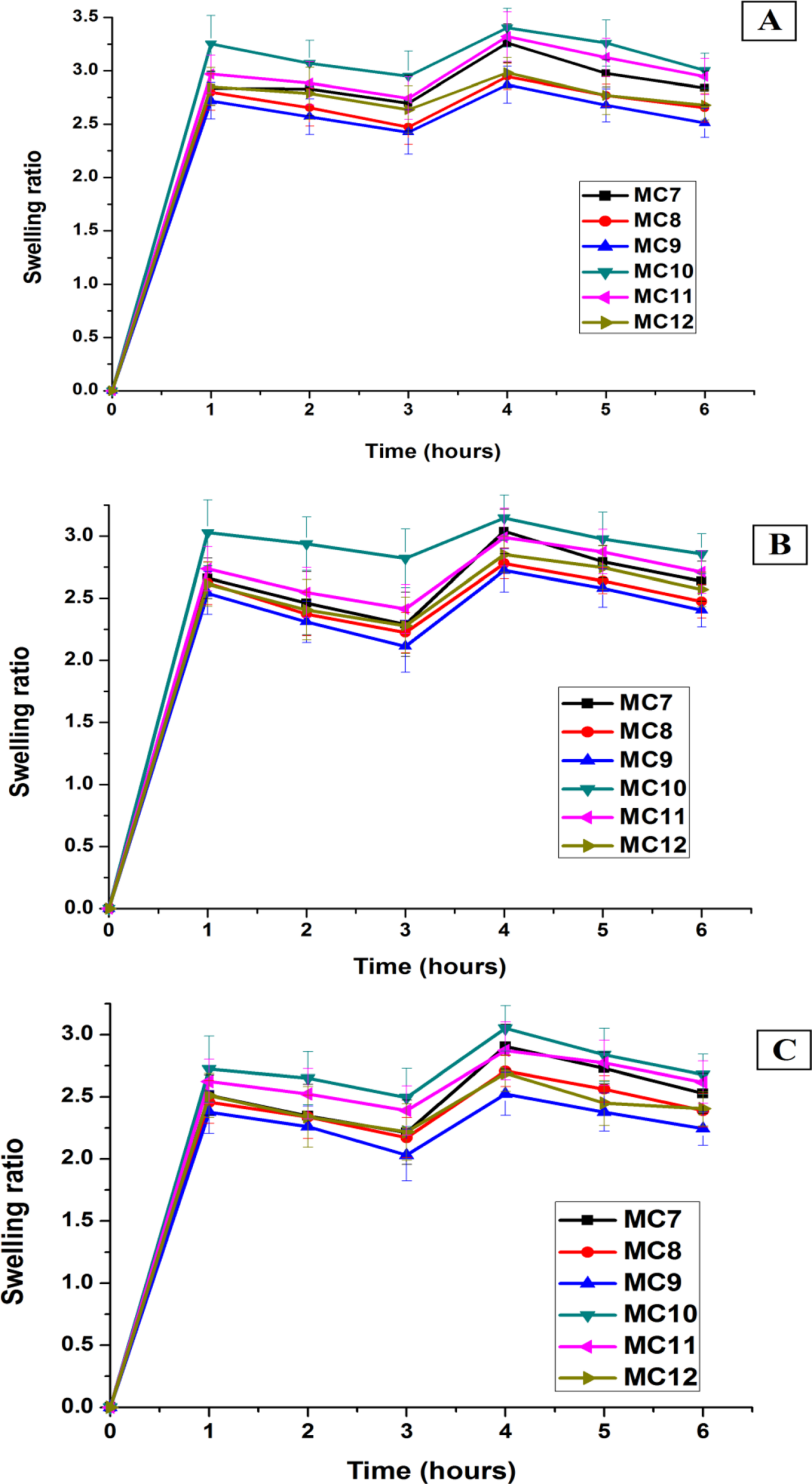
CS/PVA/PVP swelling ratio in (A) HCl, (B) NaOAc and (C) KH_2_PO_4_ buffers.

A swelling study was carried out to determine the maximum swelling capacity for each sample (Fig. 9). Because the system under consideration is a transdermal drug delivery system, swelling analysis was performed to study the effect of sweat on the prepared samples. We are interested in determining how much a sample will withstand wet conditions? Does a sample lose all of the drug present in it immediately upon swelling? Triplicate swelling tests were carried out on drug-loaded formulations in 3 distinct solutions *i.e.*, buffers with pH values of 1.2, 4.5, and 6.8 (hydrochloric acid, sodium acetate, and potassium phosphate, respectively). The purpose of using these buffers is to provide a comparative study as pH 1.2 is the pH of stomach, pH 4.5 is the pH of skin and pH 6.8 is the pH of small intestine. The swelling and erosion tests are directly related to permeation and dissolution *i.e.*, the swelling and erosion behaviors affect the drug release phenomena. It was discovered that the swelling was greater in the HCl buffer than in the NaOAc and KH_2_PO_4_ buffers. This is because the OH and NH_2_ groups of chitosan are protonated in more acidic media, creating solvation sites for H_2_O molecules. The swelling ratio increased as the PVA content increased, as PVA is more hydrophilic than PVP, increasing the swelling capability of the developed films.

With a PVA : PVP ratio of 75 : 25 in HCl buffer, MC10 had the highest swelling ratio (3.25 ± 0.26). With a PVA : PVP ratio of 25 : 75, MC9 had the lowest swelling ratio (2.72 ± 0.17). Our outcomes were in agreement with those of Chen *et al.* who compared our findings to those in the literature. They found that PVA films have a swelling of approximately >400%, while the swelling of PVP films was found to be approximately <400%.^56^ Swelling has been shown to be greater in samples lacking nanoclay.^57^

The results showed that all the samples were stable enough, and none of them decomposed during the experiment.

### 3.8. Erosion studies

Sweat on human skin causes swelling of the applied transdermal patch. When sweat is released from the patch, the drug is also released. This phenomenon of drug release is called erosion. To ascertain the rate at which the medication is released onto the skin from the sample, an erosion study was conducted. Buffers with pH values of 1.2, 4.5, and 6.8 (hydrochloric acid, sodium acetate, and potassium phosphate, respectively) were used to perform triplicate erosion experiments on the drug-containing samples (Table 2). In their study on chitosan–PEG nanocomposites for piroxicam–β-cyclodextrin delivery, Gilani *et al.* discovered that buffer with a pH of 1.2 results in the greatest amount of erosion,^28^ which changes with the PVA : PVP ratio. The erosion rate increased with increasing PVP content or decreasing PVA concentration.

**Table 2:** Erosion studies of chitosan–PVA–PVP nanocomposite films

Sample code	Percent erosion (% ± standard deviation)		
	HCl buffer	NaOAc buffer	KH_2_PO_4_ buffer
MC7	72.38 ± 0.09	71.12 ± 0.15	71.25 ± 0.28
MC8	73.78 ± 0.32	72.78 ± 0.14	72.29 ± 0.28
MC9	74.30 ± 0.32	73.96 ± 0.15	73.30 ± 0.24
MC10	74.01 ± 0.40	71.75 ± 0.54	72.77 ± 0.38
MC11	75.89 ± 0.23	73.59 ± 0.23	74.14 ± 0.27
MC12	76.08 ± 0.15	73.78 ± 0.21	74.48 ± 0.51

The erosion results showed a minor dependence on pH for the different formulations, but the erosion increased for the non-nanoclay samples, which indicates the negative role of the nanoclay on drug erosion. An increase in the PVP concentration with decreasing PVA concentration enhances erosion, which indicates a real change in the structure of the formulation. This confirms the conclusion achieved from the different characterization techniques.

### 3.9. Dissolution

**Fig. 10:**
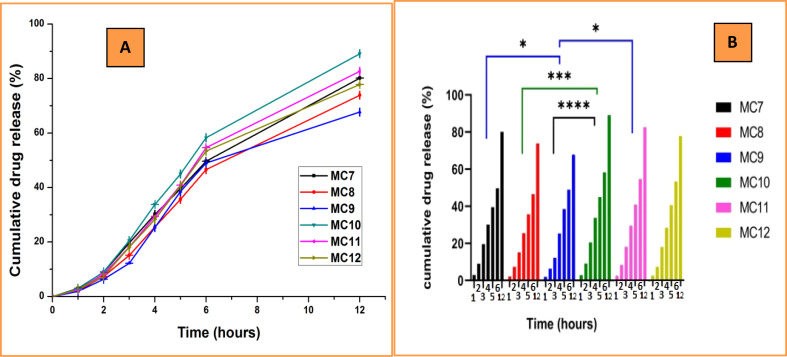
(A) *In vitro* tramadol release in MC7–MC12 (B) Dunnett's test (*n* = 3) (*p* value: *p < 0.05, ***p = 0.0005 and ****p < 0.0001).

The maximum extent of drug release for each sample was measured in dissolution analysis. This approach helps to determine the role of the sample composition of tramadol HCl release. A triplicate experiment was performed in beakers in potassium phosphate buffer (pH 6.8) to calculate the percentage of medication release in the samples at intervals of 1, 2, 3, 4, 5, 6, and 12 hours (Fig. 10). With an increase in PVA concentration or a decrease in PVP content, drug release increases. Therefore, PVP serves the purpose of controlled release. A total percent drug release of 80.14 ± 0.00351% was observed for the nanoclay-containing sample MC7 with a PVA : PVP ratio of 75 : 25. Moreover, MC9 with a PVA : PVP ratio of 25 : 75 had a total drug release percentage of 67.67 ± 0.00850%. The cumulative percent drug release for the samples without nanoclay was 89.11 ± 0.00800% for MC10 with a PVA : PVP ratio of 75 : 25 and 77.85 ± 0.00400% for MC12 with a PVA : PVP ratio of 25 : 75. Chen *et al.* reported that PVA films showed a greater percentage of cumulative release than PVP films.^56^ The drug release of MC7, MC8, and MC9 (nanoclay-containing formulations) was lower than that of MC10, MC11, and MC12, indicating that nanoclay retards drug release.^57^

The release of the drug from the formulations increased in the non-nanoclay samples, which indicates the negative role of the nanoclay on drug release. This agreed with the role of nanoclay in decreasing the swelling of nanoparticles, resulting in the path elongation of the water molecules, which was attributed to the penetration of the water molecules into nanoclay.^57^ An increase in the PVA concentration with decreasing PVP enhances drug release, possibly because of the weak attachment of the drug to the blend with a high PVA concentration. The time-rate release indicated good efficacy of the formulations for use as a tramadol delivery system because tramadol can be delivered in a continuous manner; hence, it will be available in the body for a long time and in minor quantities. This represents a health development and an economic achievement by optimizing the quantity of tramadol and the retention period of the drug inside the body.

### 3.10. Permeation

**Fig. 11:**
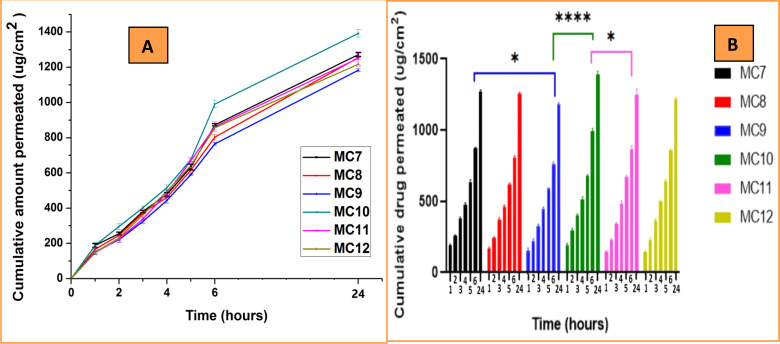
(A) Comparison of tramadol permeation in MC7–MC12 (B) Dunnett's test (*n* = 3) (*p* value: *p < 0.05 and ****p < 0.0001).

The passage of a drug through the skin is termed permeation. Permeation analysis was performed to determine the dissolution rate through human skin. Here, rat skin is used as a substitute for human skin. To determine the dissolution rate through rat skin at intervals of 1, 2, 3, 4, 5, 6, and 24 hours, a triplicate permeation experiment was carried out in potassium phosphate buffer (pH 6.8) (Fig. 11).

The findings demonstrate that when the PVA concentration increases, permeation also increases. The cumulative drug permeation of the nanoclay-containing materials MC7 with PVA : PVP of 75 : 25 and MC9 with PVA : PVP of 25 : 75 was 1271.69 ± 11.82 μg cm^−2^ and 1183.25 ± 8.46 μg cm^−2^, respectively. The cumulative drug permeation for the samples without nanoclay was 1392.01 ± 21.85 μg cm^−2^ for MC10 with a PVA : PVP ratio of 75 : 25 and 1217.56 ± 13.39 μg cm^−2^ for MC12 with a PVA : PVP ratio of 25 : 75. Valenta *et al.* studied the permeation behavior of progesterone from polyvinyl pyrrolidone (PVP), polymethacrylate (PMA), and polyvinyl alcohol (PVA) transdermal systems through excised rat skin and found maximum progesterone permeation with the PVA system.^58^ The fact that samples without nanoclay had a greater permeation rate than those with nanoclay shows that nanoclay slows drug release.

The above permeation results are directly dependent on the drug release results and hence confirmed these results.

### 3.11. Drug content uniformity

The drug content uniformity test helps to determine the distribution of the drug in the sample. The test was performed in triplicate on nanocomposite films containing the drug. The test patches were divided into two sets, proximity and center, which were then placed in KH_2_PO_4_ buffer (pH 6.8) and forcefully agitated until the whole drug mixed in the solution (Table 3). MC7 (96.09 ± 0.31%) demonstrated the highest encapsulation efficiency, whereas MC11 had the lowest encapsulation efficiency (90.56 ± 0.34%). The amounts were almost the same in both the center and the surrounding area, showing that the drug particles were dispersed evenly throughout the created nanocomposites.

**Table 3:** Percent of tramadol HCl loaded in the MC7–MC12 formulations

Code	Tramadol HCl contents in the center (% ± SD)	Tramadol HCl contents in proximity (% ± SD)
MC7	96.09 ± 0.31	95.48 ± 0.33
MC8	90.69 ± 0.48	90.99 ± 0.74
MC9	93.16 ± 0.51	92.60 ± 0.49
MC10	91.88 ± 0.58	92.04 ± 0.35
MC11	90.56 ± 0.34	91.51 ± 0.24
MC12	94.76 ± 0.21	93.30 ± 0.68

## 4 Conclusion

In this study, CS–PVA–PVP ternary blends were used as transdermal patches for tramadol delivery. Different physical and pharmaceutical analyses were carried out to test the formulations. Some interesting findings of the study are listed here. The prepared samples are thermally stable enough. They withstand temperatures as high as 500 °C. The samples are crystalline and have no gaps or cracks. The rates of swelling, erosion, permeation and dissolution were all impacted by the PVA : PVP ratio shift. With increasing PVA concentration, the swelling, dissolution, and permeation rates all increased. Nanoclay retarded the rate of tramadol release in the nanocomposite films. As the PVA concentration increased, erosion decreased.

The produced nanocomposites could be used as transdermal drug delivery materials based on their characteristics. This represents a health development and an economic achievement by optimizing the quantity of tramadol and the retention time of the drug inside the body.

## Ethical statement

All study protocols were approved by the Departmental Research Ethics Committee of COMSATS University Islamabad with approval number 893/CUI/PHM-2022.

## Author contributions

Conceptualization, Sadullah Mir; formal analysis, Mohsin Ali; funding acquisition, Mohsin Kazi; investigation, Mohsin Ali; methodology, Mohsin Ali; project administration, Obaid-Ur-Rahman Abid; software, Mohsin Ali; validation, Leonard Atanase; writing – original draft, Mohsin Ali; writing – review and editing, Mohsin Kazi.

## Conflicts of interest

There are no conflicts to declare.

## Supplementary Material
